# Can Biological Drugs Diminish the Risk of Sarcopenia in Psoriatic Patients? A Systematic Review

**DOI:** 10.3390/life12030435

**Published:** 2022-03-16

**Authors:** Zuzanna Piętowska, Danuta Nowicka, Jacek Szepietowski

**Affiliations:** Department of Dermatology, Venereology and Allergology, Wroclaw Medical University, 50-368 Wroclaw, Poland; zuzia.pietowska@gmail.com (Z.P.); jacek.szepietowski@umw.edu.pl (J.S.)

**Keywords:** sarcopenia, psoriasis, psoriatic arthritis, muscle loss, body composition, drugs intake, biological therapy

## Abstract

Sarcopenia and psoriasis are different inflammatory diseases that share common comorbidities (e.g., cardiovascular diseases, metabolic syndrome, obesity, autoimmune diseases, depression). Psoriasis is a dermatosis involving the skin, joints, and nails. Its estimated prevalence is 2–4%, and the possibility of progression to psoriatic arthritis reaches 6–42%. Sarcopenia is defined as reduced muscle strength, muscle quantity, and physical performance due to non-ageing related causes. It affects up to 10% of the general population. We conducted a review of the literature to provide up-to-date information about the risk of sarcopenia in psoriasis and to identify risk factors that increase this risk. The search of the literature allowed us to include 51 publications, but only five cross-sectional studies provided quantitative results on the rates of sarcopenia in psoriasis. The prevalence of sarcopenia in psoriasis varied from 9.1% to 61.7%. This wide range was caused by different definitions of sarcopenia and different cut-off values across studies. Prognostic factors include lean mass and fat mass. Further research based on the European Working Group on Sarcopenia in Older People guidelines is required. Such studies should include not only muscle mass and strength but also other factors that may influence the occurrence of sarcopenia and inflammatory markers.

## 1. Introduction

The term “sarcopenia” was used for the first time by Rosenberg I. in the 1980s as an age-related loss of skeletal muscle mass and important change of body composition [1]. At the turn of the decade, the definition of sarcopenia evolved and included a broader spectrum, particularly muscle wasting associated with chronic diseases, malnutrition, impairment of mobility and decreased physical activity. Moreover, it has been indicated that secondary sarcopenia must be separated from primary age-related sarcopenia [2].

The European Working Group on Sarcopenia in Older People (EWGSOP) in 2010 amended the definition of sarcopenia, emphasizing the essence of identification and healthcare of patients from the risk groups. The EWGSOP focused on muscle function and strength examination in contrast to the prior standpoint, muscle mass loss detection only [3,4,5]. At the second meeting in 2018 (EWGSOP2), the guidelines were revised, and the four insights of the contemporary definition of sarcopenia were enunciated: non-ageing related causes, low muscle strength as the main determinant, difficulty measuring muscle quantity and quality in clinical practice, and complexity of diagnosing and treatment [6]. EWGSOP2 presented a practical approach to diagnosing sarcopenia by using selected validated tests and tools from a variety of possibilities [6,7,8]. The EWGSOP recommends the use of the SARC-F questionnaire [9] as a screening tool, which has a high specificity in predicting low muscle strength [10]. It is based on the subjective assessment of the patient and is a low-cost, prompt and convenient screening method. It has been translated and validated in many languages, including Polish [11,12]. EWGSOP2 summarized the measurements of sarcopenia’s essential parameters: muscle strength, muscle quantity and physical performance. Muscle strength is estimated by grip strength, measured by a calibrated hand dynamometer and correlates with strength in other extremities [13,14]. Muscle quantity is given as appendicular skeletal muscle mass or total body skeletal muscle mass. It is measured using diverse techniques, for instance, magnetic resonance imaging, computed tomography, dual-energy X-ray absorptiometry or bioelectrical impedance analysis [14,15]. Furthermore, physical performance is estimated by gait speed, the Short Physical Performance Battery [16], the Timed-Up and Go test [17] and the 400 m walk test. The EWGSOP recommends starting a diagnostic process with evaluation of physical performance and measurement of the severity of sarcopenia, which have prognostic values for adverse health outcomes related to sarcopenia [18,19].

Simultaneously, another thesis was created by the Asian Working Group for Sarcopenia (AWSG) as a consensus, where different cut-off points were adapted to the Asian population [20]. In 2019, it was updated with a new diagnostic algorithm, protocols and criteria [21]. Likewise, their definition of sarcopenia was developed by the International Working Group on Sarcopenia [22] and the Foundation for the National Institutes of Health [23]. The milestone was defining sarcopenia as a disease in 2016 by the World Health Organization (WHO), with its individual ICD-10-CM code: M62.84 [24,25]. This should lead to the improvement of the diagnostic approach, detection rate and development of treatment guidelines of sarcopenia.

Various sources indicate a different prevalence of sarcopenia in the population. A systematic review and meta-analysis from the University of Medical Sciences in Tehran reported a frequency of 10% both for women and men [26], whereas according to the old definition, these values could even reach 40% [27]. Furthermore, the prognosis of the growing prevalence of sarcopenia during the next 30 years is extremely alarming and makes this disease a major public health issue [28].

Sarcopenia has extremely serious far-reaching adverse health outcomes, especially in elderly populations such as an elevated risk of falls and fractures [29], a greater number of hospitalizations, increased disability, and functional decline [30], and is accompanied by deterioration of cognitive functions [31] and even depression [32]. It also significantly decreases quality of life (QoL) [33,34]. Moreover, sarcopenia is associated with broadly defined pathological metabolic changes, such as the incremental risk of insulin resistance, diabetes mellitus, osteoporosis, escalated lipolysis and accumulation of free fatty acids [35,36,37,38]. The pitfall of sarcopenic obesity, which arose in recent years, seems to be meaningful. This state is characterized by a reduction in muscle mass and muscle quality with a simultaneous increase in adiposity. Some pathogenetic pathways in sarcopenia and obesity overlap, giving rise to the term sarcopenic obesity [39]. This condition is associated with an augmented metabolic load, which has a significant influence on cardio-metabolic and physical facilities, up to and including death [40,41]. However, any international consensus concerning sarcopenic obesity has not yet been developed. The creation of guidelines would allow for a better understanding of this new public health problem, as well as for decreasing the incidence of cardiovascular diseases and reducing mortality [42,43,44]. Despite the clinical importance of sarcopenia, this condition remains underrecognized and undertreated in routine clinical practice. To draw attention to this health issue, sarcopenia should become a primary topic of medical and political debate because of its enormous impact on morbidity, mortality and healthcare expenditure.

Psoriasis is a chronic inflammatory genetic disease involving the skin, joints and nails. In erythrodermic psoriasis, inflammatory lesions such as erythema and exfoliation of the skin can cover over 90% of the body area. Psoriasis is the most common skin disease, but it is also an intensely stigmatizing and discriminating dermatosis that strongly interferes with QoL and affects all its aspects, going far beyond skin symptoms. New concepts of psoriasis as a systemic, immune-mediated disorder explain its chronicity and cooccurrence of a wide range of comorbidities. The etiopathogenesis of this dermatosis is abstruse and is still not fully understood; however, understanding the role of the immune-mediated processes help to manage this chronic disease [45]. It has been known for many decades that psoriasis imposes on patients a severe psychological burden comparable to cancer, depression or diabetes [46]. In western nations, the prevalence of psoriasis is estimated at 2–4% of the population, a percentage depending on multiple genetic and environmental factors [47,48,49].

Psoriatic arthritis (PsA) is one of the many seronegative spondyloarthropathies related to psoriasis. The studies inform about the risk of developing psoriatic arthritis in patients with psoriasis to be between 6% to even 42% [50]. Therefore, it is important to diagnose and treat PsA promptly, as it can lead to the development of progressive joint damage due to chronic inflammation, resulting in disability and severe physical, social and mental impairment. For this reason, it seems appropriate to encourage physicians to actively seek symptoms of PsA in every patient with psoriasis [51,52]. These two conditions also share other numerous comorbidities, such as cardiovascular diseases, metabolic syndrome, obesity, autoimmune diseases, depression and even an increased risk of committing suicide [53].

Chronic inflammation, with all its consequences such as inflammatory arthritis, generalized atherosclerosis, myocardial infarction, stroke and cardiovascular death, seems to be important in psoriasis [54,55,56,57,58]. In this condition, T-helper cells (Th1, Th17, Th22) after their activation and proliferation produce a number of pathogenic cytokines: tumour necrosis factor-α (TNF-α), interferon (IFN)-γ, and interleukin (IL)-2, IL-6, IL-17A, IL-17F, IL-22, IL-26 [59,60], which is backed by a well-documented strong relationship between obesity and psoriasis. The pathophysiological cause of both conditions is tumour necrosis factor-mediated chronic inflammation and thus a cascade of immunological and metabolic effects. This explains why patients with this chronic inflammatory disease report clinical improvement, relief of symptoms of psoriasis and weight loss after the introduction of physical activity and a low-calorie diet with the aim to reduce oxidative stress [45,61]. Adipose tissue is one of the largest organs in the endocrine system, producing pro-inflammatory cytokines (TNF-α, IL-6, MCP-1), adipokines and hormonally active proteins (adiponectin, leptin, resistin, apelin, visfatin) [62]. It is suspected that TNF-α in obesity comes from adipose tissue macrophages and, in psoriasis, from activated T cells. Research shows that adipokines, both leptin and resistin, which enhance the production of proinflammatory cytokines (e.g., TNF-α), may be involved in the pathogenesis of psoriasis [63,64,65]. Systemic inflammation, fuelled by pro-inflammatory cytokines and adipokines, leads to a progression of endothelial cell damage, atherogenesis, and as mentioned above, to an imminent development of major adverse cardiovascular events [57,66,67,68]. The inflammatory background of psoriasis has prompted the use of agents that can target a specific part of the immune system, also called biologic therapy or simply biologics. These agents are generally well tolerated and are effective in the prevention of further damage to the joints [50].

Taking into account the characteristics of psoriasis and sarcopenia, we conducted a review of the literature to summarize data on the risk of sarcopenia in patients with psoriasis. To this aim, we investigated and summarized the current knowledge on risk factors, including but not limited to idiosyncratic risk factors, obesity, specific treatment for psoriasis or cessation of treatment that have the potential to increase the risk of developing sarcopenia in psoriatic patients.

## 2. Materials and Methods

We performed a review of the literature [69] in line with the PRISMA guidelines by searching electronic databases (MEDLINE via PubMed, EMBASE via SCOPUS) for articles of any type, published in English since 1993. Afterwards, a manual search of the references of the mentioned articles was conducted as a research supplement. The exploration was limited to human studies. The search was updated from November 2021 to January 2022.

The following medical subject heading terms were utilized for the search: “sarcopenia” AND “psoriasis” OR “psoriatic arthritis” OR “arthritis” OR “risk factors” OR “drugs intake” OR “biological therapy” OR “skin disease”, “dermatology” likewise “psoriasis” AND “muscle loss” OR “body composition” OR “obesity”.

**Figure 1:**
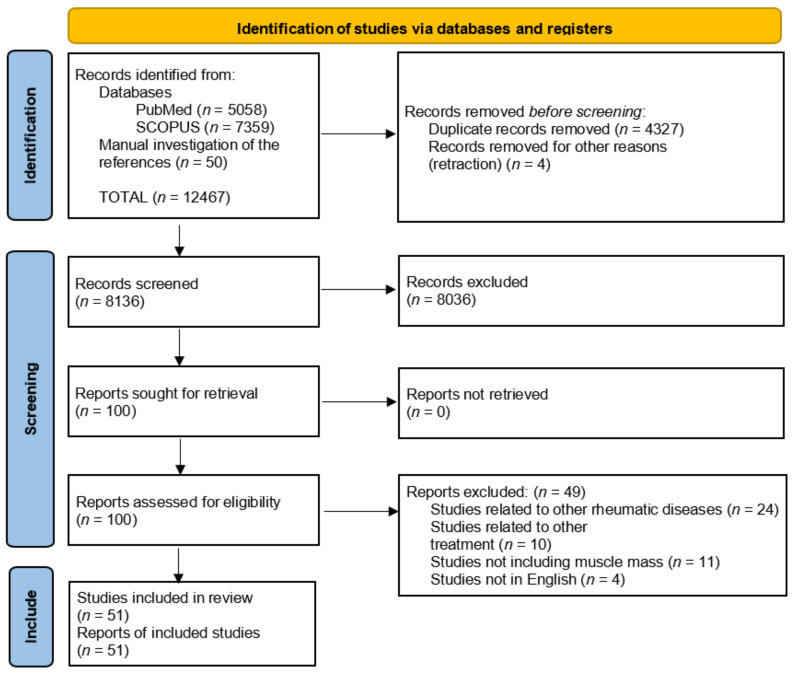
PRISMA flow diagram.

After the first screening, 100 papers were found; however, after precise review of titles, abstracts and after assessment of eligibility, 51 articles were included. Of the included studies, 5 cross-sectional studies provided quantitative results on the rates of sarcopenia in psoriasis. The process and results of the literature screening are shown in Figure 1. The results were summarized in the form of a systematic review.

## 3. Results

Only a few studies assessed the risk of prevalence of sarcopenia in psoriasis. Only one study was designed to investigate the incidence of sarcopenia in plaque psoriasis [70]. We found four studies investigating the relationship between sarcopenia and psoriatic arthritis [71,72,73,74] (Table 1).

Aguiar et al. [71] reported that 61.7% of patients with spondyloarthritis developed sarcopenia, compared to the control group, in which this ratio was 43.3% (OR = 5.23, *p* < 0.01). They showed a substantial decrease in the mean muscle mass index (MMI) in the study group (7.65 ± 0.98 vs. 8.25 ± 0.92, *p* = 0.001). The study showed no significant difference in MMI between patients with psoriatic arthritis and ankylosing spondylitis (*p* = 0.323). There was no significant correlation between MMI and the Disease Activity Score-28 (DAS28) for the peripheral form and the Bath Ankylosing Spondylitis Disease Activity Index (BASDAI) for the axial form. For both forms of the disease, MMI did not correlate with function indices such as the Health Assessment Questionnaire (HAQ) and the Bath Ankylosing Spondylitis Functional Index (BASFI), as well as radiographic damage measured with the modified Stoke Ankylosing Spondylitis Spinal Score (mSASSS). The separate analysis showed that in male patients with spondyloarthritis, there was a statistically significant moderate negative correlation between MMI and BASDAI and BASFI. However, the study did not refer to muscle function, but only to the reduction in muscle mass.

Barone et al. evaluated patients with rheumatic diseases, including psoriatic arthritis. In total, 168 adult patients were enrolled, after the previous exclusion of 36 patients not meeting the inclusion criteria, such as, for example, obesity. PsA was diagnosed in 70 patients. The diagnosis of sarcopenia was made based on the assessment of muscle mass and strength. MMI was assessed anthropometrically, and using bioelectrical impedance analysis, handgrip strength was also taken into account. Barone et al. did not show a statistically significant difference in the incidence of sarcopenia in the studied groups of patients with rheumatoid arthritis, psoriatic arthritis and ankylosing spondylitis. They assessed the incidence of sarcopenia and presarcopenia in the entire cohort at the levels of 20.8% and 20.2%, respectively. In the group of PsA patients, the incidence of sarcopenia remained at a similar level, while that of presarcopenia was 25.7% (*p* = 0.006). Moreover, they showed a link between the incidence of sarcopenia and disability, C-reactive protein (CRP) level and age. However, this study did not include a control group [72,75].

Krajewska-Włodarczyk et al. examined a group of 51 women with PsA, aged over 50 years. Muscle mass was assessed by BIA. In addition, the following indicators were estimated: appendicular lean mass (ALM), the skeletal muscle mass index (SMI), and the Timed Ap and Go Test (TUG). The prevalence of sarcopenia was associated with the method used for the assessment. SMI was significantly lower in the PsA group compared to the control group, while ALM showed no such correlation. Depending on the method used, the percentage of women with sarcopenia and PsA ranged from 13.7% to even 43.1%. However, the relevant *p* value was not reported. In this group, a statistically significant correlation between sarcopenia and bone demineralization was also demonstrated. However, no correlation was observed between a decrease in muscle mass and the indicators of inflammation and disease activity [73].

The last study of Tournadre et al., determining the incidence of sarcopenia in the PsA group, did not have a properly selected control group [75]. They reported sarcopenia in 9.1% of patients based on muscle mass and function criteria. This value did not change when considering the loss of muscle mass alone [74].

The only study on the incidence of sarcopenia in plaque psoriasis was the work of Chen et al. [70], in which no significant association was found between psoriasis and sarcopenia (OR = 0.51, *p* = 0.136). This disease was associated with myosteatosis based on skeletal muscle radiodensity (OR = 3.16, *p* < 0.001) and on intermuscular adipose tissue (OR = 1.76, *p* = 0.037), calculated based on the unenhanced cross-sectional chest-computed tomography images. However, in this study, muscle function and inflammatory markers (CRP, IL-6) were not taken into consideration. Furthermore, the study enrolled relatively young patients aged 44.7 years. PsA patients were excluded from the study.

## 4. Discussion

Our study showed that evidence on the prevalence of sarcopenia in patients with psoriasis is limited. In the identified studies, it ranged from 9.1% to 61.7%. Possible causes of differences in the results include different definitions of sarcopenia and different cut-off values across studies. The need to standardize both the definition and diagnostic criteria of sarcopenia seems to be urgent because sarcopenia is directly related to increased mortality among patients [76,77,78]. The above-mentioned studies suggest that there is a potential risk of an increased incidence of sarcopenia in patients with psoriasis. However, the data are ambiguous, and it is impossible to estimate the exact value of this risk. Due to the burden of sarcopenia and its health consequences, it seems necessary to investigate this topic, especially because the relationship between sarcopenia and other autoimmune, rheumatic and metabolic diseases has been confirmed in paediatric patients [79,80,81,82,83].

Beenakker et al. [84] pointed out a possible relationship between age-related inflammation and sarcopenia. They reported the occurrence of premature loss of muscle strength measured by handgrip strength in patients with elevated activity of the chronic inflammatory disease. They confirmed the results of previous studies that had shown the adverse effect of pro-inflammatory cytokines on muscle mass and strength, which may translate into a direct risk of developing sarcopenia [85,86]. IL-6 and other cytokines such as TNF-α and IL-1β, which have a profound effect on energy and protein metabolism, were referred to by Roubenoff as “sarcoactive” [87]. These cytokines play an important role in the pathogenesis of many immune-mediated diseases, including psoriasis [45,88,89,90,91]. These associations may implicate that the inflammatory response promotes sarcopenia as confirmed by Bhatnagar et al. who presented data on the effects of TNF- α on muscle wasting by modifying numerous molecular pathways, including NF-kB and Notch1 signalling, cellular apoptosis, and myocyte proliferation and differentiation [92]. The involvement of these cytokines in age-related sarcopenia and ageing of the healthy population has also been described by Schaap et al. [93].

The pathogenesis of psoriasis is closely related to the expression of TNF-α [94]. Anti-TNF-α therapy is effective and results in rapid clinical responses in psoriasis patients, making these drugs one of the first-line therapies [95]. According to Campanati et al., psoriasis is associated with a disturbance of the balance between anti-inflammatory and pro-inflammatory adipokines; the difference slightly decreases after the use of TNF-α blockers but does not normalize [96]. However, numerous studies show that this treatment has a negative effect on body weight and body mass index (BMI) due to an increase in the fat-free mass caused by anti-TNF-α therapies [75,97,98]. Moreover, special attention should be paid to the potential side effects of golimumab, infliximab, etanercept and adalimumab [99]. This is an important issue that must be taken into account when selecting a treatment line because patients with psoriasis have an elevated risk of comorbidities such as obesity, cardiovascular diseases, metabolic syndrome, diabetes and depression [55,56,57,100,101,102]. Furthermore, weight gain directly increases the risk of the aforementioned diseases. This could potentially be associated with the development of sarcopenia and sarcopenic obesity; nonetheless, this problem requires further investigation due to the results of the research by Renzo et al. [103]. The study showed that suppression of TNF-α-mediated pathways in patients with psoriasis vulgaris and PsA results not only in an increase in fat mass but also in lean mass. They concluded that TNF-α blockers and the reduction of TNF-α and IL-6 may be the beginning of a complex relationship between muscle mass and fat mass. Moreover, their research on −174G/C polymorphism of the IL-6 gene and obesity in relation to the therapeutic response to TNF-α blockers showed that the polymorphism can be considered a risk factor in the prognosis of psoriasis treatment. This polymorphism and obesity predicted a poor response to TNF-α blockers [104], which makes the selection of the right therapy for psoriasis even more difficult in terms of muscle mass, fat mass, other risk factors and other variables.

Another study by Kofoed et al. [105] investigated the dependence of anti-TNF-α therapy on body composition and insulin sensitivity. They confirmed that the increase in body weight during this therapy was associated with increased trunk fat, but they were unable to confirm a significant effect of this therapy on insulin sensitivity. Martínez-Abundis et al. [106] reported similar results. Opposite results were presented by Marra et al. [107], who reported dilution in insulin resistance after 24 weeks of etanercept treatment. These studies emphasize that an in-depth study must be conducted to understand problematic relationships among sarcopenia, obesity, inflammation, metabolic disturbances and psoriasis.

A meta-analysis by Patsalos et al. suggests that the side effects of TNF-α blocker therapy, such as an increase in lean and fat mass, could potentially be used to treat cachexia caused by malignant neoplasms and anorexia nervosa [99]. This fact may influence the management of the patient with many comorbidities.

Psoriasis and obesity are both chronic inflammatory diseases, both of which share the same inflammatory mediators. The pro-inflammatory adipokines overproduced in obesity and metabolic syndrome stimulate dermatitis in psoriasis patients, and pro-inflammatory cytokines in psoriasis modulate adipokine secretion and lead to metabolic dysregulation, atherosclerosis, insulin resistance and inflammation stimulation. In these states, inflammatory mediators mutually stimulate each other and activate subsequent pathological pathways [66,108,109,110]. Among the cytokines secreted by cells infiltrating adipose tissue is IL-17. Additionally, the adipocytes themselves produce leptin and adipokine-stimulating Th1 lymphocytes to produce the mentioned cytokine. As we know, this cytokine is closely related to the pathogenesis of psoriasis and other inflammatory diseases, which confirms the connection between psoriasis and obesity [111,112,113,114,115,116,117]. Chen et al. [70] showed an independent significant relationship between psoriasis and myosteatosis. They did not show a dependence on sarcopenia; however, they did not use the EWGSOP criteria for sarcopenia, which could have affected the study results.

Blake et al. [75] emphasized that body composition profiles were related with anti-IL-12/23 and PDE4 (apremilast) inhibitors treatment compared to TNF-α blockers. It seems reasonable to target pro-inflammatory agents such as IL-17 or IL-12/23 to achieve concomitant benefits in addition to acting on psoriasis per se.

Piros et al. proved the relationship of IL-17 blockers with the improvement of inflammatory and lipid parameters after treatment. However, they did not observe changes in body composition parameters measured with bioelectrical impedance analysis. They emphasized the beneficial effect on reducing eventual comorbidities, especially cardiovascular and metabolic diseases [118].

Galluzzo et al., in their work, presented optimistic results concerning anti-IL-12/23 agents and body composition, and more specifically, an increase in phase angle and body cell mass and a decrease in BMI and fat mass in patients with psoriasis. Body cell mass is a metabolically active part of the body, the “protoplasm”, while the phase angle is associated with body cell mass and integrity of the cell membrane [119].

Using BIA and determination of a simple biophysical parameter phase angle, Barrea et al. [120] showed the effect of the relationship of inflammation associated with psoriasis and metabolic syndrome on muscle mass. This parameter is treated as an indicator of “cellular health” [75]. The higher its value, the better the integrity of cell membranes and their broadly understood functioning. In the population of healthy people, its value decreases with age; in disease states, this parameter decreases due to inflammation and other factors related to the pathological state [121]. In recent years, this parameter has been presented as a good prognostic tool in medical conditions such as cancer and diabetes mellitus [122,123]. This parameter could be incorporated into general medical practice to assess patients’ QoL, clinical severity and muscle deficit in psoriasis as well.

Data on the influence of biological drugs on body composition and the development of sarcopenia are scarce. This topic requires attention because the consequences of sarcopenia are enormous both for the patient and for the entire healthcare system. A faster and more efficient diagnosis of sarcopenia would improve QoL and life expectancy of patients and would reduce the mortality associated with psoriasis.

## 5. Conclusions

Data on the risk of sarcopenia in psoriasis are scarce and incomplete. The relationship between these two conditions is highly probable. Examination of body composition, including lean mass and fat mass, and their quality as well as distribution, should be routine in clinical practice. These parameters are considered important prognostic factors in the treatment of psoriasis. Further studies are required on large groups of patients with standardized inclusion and exclusion criteria based on the EWGSOP guidelines relating not only to muscle mass but also to strength, including other factors that may influence the occurrence of sarcopenia such as disease duration, treatment, activity and severity of the disease, age, gender, physical activity, and supply of nutrients. It is also necessary to investigate associations among inflammatory markers (IL-6, TNF-α, CRP), myosteatosis, blood lipid levels, adiposity, osteoporosis or osteopenia, bone demineralization and other comorbidities.

## Figures and Tables

**Table 1:** Prevalence of sarcopenia in psoriatic arthritis (PsA): summary of the collected research.

Author	Country, Year	Study Design	Sarcopenia Prevalence	*p* Value	Participants	Sarcopenia Assessment
Aguiar et al. [71]	Portugal, 2014	Cross-sectional	61.7%	0.001	60 M, Caucasian, 45.5 ± 13.4 yo	MMI (Lee’s equation)
Barone et al. [72]	Italy, 2018	Cross-sectional	20.0%	NS	70 M/W, Caucasian, 51.6 ± 8.8 yo	MMI (BIA) + HS
Krajewska-Włodarczyk et al. [73]	Poland, 2017	Cross-sectional	13.7% 49.0% 43.1%	NS <0.001 -	51 W, Caucasian, 65.6 ± 5.9 yo	ALM (BIA), SMI (BIA), SMI, TUG
Tournadre et al. [74]	France, 2017	Cross-sectional	9.1% 9.1%	0.009 -	148 W, 54.6 ± 11.0 yo	SMI (DXA), SMI + HS

ALM, appendicular lean mass index; BIA, bioelectric impedance analysis; DXA, dual-energy X-ray absorptiometry; HS, handgrip strength; M, men; MMI, muscle mass index; NS, not significant; SMI, skeletal muscle mass index; TUG, Timed Up and Go Test; W, women; yo, years of age.

## Data Availability

All data are included in the manuscript.
